# Regulation of the heterochromatin spreading reaction by *trans-*acting factors

**DOI:** 10.1098/rsob.230271

**Published:** 2023-11-08

**Authors:** Bulut Hamali, Ahmed A. A. Amine, Bassem Al-Sady

**Affiliations:** ^1^ Department of Microbiology and Immunology, University of California San Francisco, San Francisco, CA 94143, USA; ^2^ The G. W. Hooper Foundation, San Francisco, CA 94143, USA; ^3^ College of Dentistry, The Ohio State University, Columbus, OH, USA

**Keywords:** heterochromatin spreading, gene silencing, *trans*-acting factors, polycomb, Suv39, Silent Information Regulator

## Abstract

Heterochromatin is a gene-repressive protein–nucleic acid ultrastructure that is initially nucleated by DNA sequences. However, following nucleation, heterochromatin can then propagate along the chromatin template in a sequence-independent manner in a reaction termed spreading. At the heart of this process are enzymes that deposit chemical information on chromatin, which attracts the factors that execute chromatin compaction and transcriptional or co/post-transcriptional gene silencing. Given that these enzymes deposit guiding chemical information on chromatin they are commonly termed ‘writers’. While the processes of nucleation and central actions of writers have been extensively studied and reviewed, less is understood about how the spreading process is regulated. We discuss how the chromatin substrate is prepared for heterochromatic spreading, and how *trans-*acting factors beyond writer enzymes regulate it. We examine mechanisms by which *trans-*acting factors in Suv39, PRC2, SETDB1 and SIR writer systems regulate spreading of the respective heterochromatic marks across chromatin. While these systems are in some cases evolutionarily and mechanistically quite distant, common mechanisms emerge which these *trans-*acting factors exploit to tune the spreading reaction.

## Graphical abstract

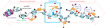

## 1. Introduction

Heterochromatin is a gene-repressive chromatin structure that has been visualized cytologically for over a century. The name was coined by Emil Heitz and describes chromosomal domains that remain condensed throughout the cell cycle [1]. We have learned much about heterochromatin over the century, yet its behaviours remain important to explore, given its central role in the eukaryotic cell: constitutive heterochromatin shapes the normal functioning of the genome, while facultative heterochromatin, which can change across lineages, directs normal development in multicellular organisms. In some ways, both types of heterochromatin, but especially facultative heterochromatin, are formed by a process similar to the activation of transcription: DNA sequences dictate the local recruitment of repressive factors [2,3]. The field terms those ‘nucleation sites’ rather than promoters and enhancers. What has remained intriguing about heterochromatin is its ability to propagate itself outwards from such DNA-sequence encoded signals for significant distances along the chromosome. This sequence-independent extension of heterochromatin is a process referred to as ‘spreading’ [4] and encompasses both the chromosomal extension of function and structure (i.e. gene expression and the associated changed chromatin state and protein composition). This process is highly dosage-sensitive to key regulators, which was for example observed for subtelomeric silencing by the Silent Information Regulator (SIR) proteins in *S. cerevisiae* [5–7], and position effect variegation in *D. melanogaster* [8]. At the heart of nucleation and spreading is the action of the central enzymes (writers) that deposit repressive chromatin marks that signal the assembly of the gene repressive heterochromatic structure. Much has been written about the properties of the writer enzymes, here we want to explore how *trans-*acting factors enable and control this process by writers across systems and examine some unique and shared characteristics.

## 2. General substrate requirements for heterochromatin spreading

**Figure 1.**
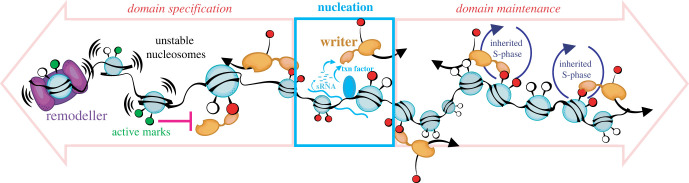
Heterochromatin spreading occurs in different chromatin environments and on substrates of different histories. ‘Writers’ are nucleated directly via transcription factors or indirectly via small RNA processes (centre). The writer then can spread on chromatin that was previously heterochromatic (domain maintenance, right), thus inheriting nucleosomes through S-phase, enabling positive feedback. The region also may be diminished in spreading-antagonizing transcriptional activities, such as in repetitive regions. The writer may also spread into a region de novo (domain specification, left)*,* where it has to contend with multiple antagonizing activities, including nucleosome destabilization and inhibitory chromatin marks.

While the heterochromatin systems we will discuss below do have some differences in their requirements for spreading away from DNA-encoded nucleation sites, there are some universal features of the substrate, chromatin, that either encourage spreading or hinder it. On a first level, it is useful to think of heterochromatin spreading as a reaction that can occur to re-establish the initial stage (domain maintenance), or, to establish a repressed domain for the first time (domain specification, we avoid the term establishment as this typically refers to the first nucleation event; figure 1). The chromatin environments in these two contexts are rather different: in the case of maintenance, this occurs in regions that have already been repressed and may be additionally gene-poor, such as constitutive heterochromatin. Here, RNA polymerase is typically less active (although, for co-transcriptional gene silencing, some transcription does occur). Nucleosomes bearing repressive marks, such as methylation at H3 lysine 9 or 27, are partially inherited (figure 1), which directly facilitates the re-establishment of the initial state by the writer enzymes. This is because writer enzymes exploit positive feedback encoded in those inherited methylated nucleosomes: As ‘read-write’ enzymes, the writers recognize their reaction product in the modifying subunit or other complex subunit, which facilitates further modification via a variety of mechanisms [9–13]. This type of positive feedback has long been predicted by theoretical approaches to be required for the formation of a stably repressed domain (e.g. [14]).

**Figure 2.**
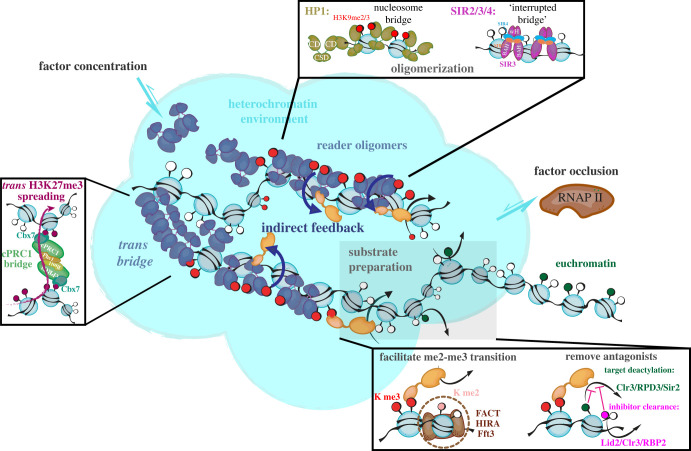
Mechanisms by which *trans*-acting factors promote spreading. Central *trans*-acting factors produce a heterochromatin niche, either via condensates, or subnuclear localization, where heterochromatin factors are enriched, and transcription-activating factors are de-enriched. These central *trans*-acting factors direct feed into the positive feedback of the writer and stabilize the chromatin substrate via oligomerization, which also promotes spreading via the positive feedback. TOP BOX: HP1 and Sir3 are examples of two proteins that cross-bridge nucleosomes via their oligomeric properties. HP1 domains highlighted: chromodomain (CD, me2/3 binding and oligomerization), chromo shadow domain (CSD, dimerization). Sir3 domains highlighted: winged helix (wH, di- and oligomerization), bromo-adjacent homology (BAH, nucleosome binding). LEFT BOX: Long-range spreading (trans-spreading is enabled by canonical PRC1 (cPRC1) via its H3K27me binding and oligomeric properties. RIGHT BOX: The chromatin substrate is prepared for productive spreading by factors that stabilize nucleosomes, such as FACT, Fft3 or HIRA. This allows productive methylation to the trimethylated state, which is required for spreading and silencing in the case of Suv39 and PRC2 ‘writers’. *Trans*-acting factors also remove occluding (on the substrate lysine) and antagonizing (inhibit writer activity on substrate lysine) marks.

By contrast, a newly specified heterochromatin domain cannot inherit pre-modified nucleosomes, therefore, the initial specification by spreading does not have the opportunity to exploit this positive feedback. Moreover, the chromatin template is more hostile to heterochromatin: genes are active, and transcription can directly or indirectly abrogate spreading. It does so largely in two ways: first, destabilizing nucleosomes, or even creating nucleosome-free regions (figure 1). Just as nucleosome-free regions ‘poison’ spreading [15], unstable nucleosomes can inhibit spreading as well, especially in systems that need to reach a fully methylated state for repression and spreading (PRC2, Suv39, SETDB1; figure 2). This trimethyl state is often critical for gene silencing, for example, H3K9me2 can be permissive to transcription [16], and the trimethyl state can be instructive for positive feedback [9]. Methyl writers are thought to require stable nucleosomes to reach the fully methylated state as they are not processive for trimethylation on the nucleosome substrate, e.g. Suv39h1 [17]. The *in vivo* appearance kinetics of H3K27me3 also suggest that PRC2 is not primarily processive for the terminal state [18,19]. In a distributive mode and relatively slow kinetics, continuous residence of the target is essential for reaching trimethylation. Hence, these heterochromatin systems are sensitive to nucleosome turnover: factors that mobilize or stabilize nucleosomes antagonize and promote spreading, respectively. Second, active genes can contain chromatin marks that directly antagonize the enzyme itself. For example, H3K4 or K36 trimethylation, which mark active genes, can inhibit heterochromatic enzymes such as G9a/GLP and Suv39 [20], as well as PRC2 [21,22]. The SIR3 protein which spreads SIR heterochromatin is directly inhibited in its ability to bind nucleosomes by chromatin marks associated with gene activity [23,24] (figure 1). This antagonism can be critical to rejecting ectopic nucleation and spreading into active genes.

Another broad level of regulation is the nuclear compartment. It has been known for a long time that hetero- and euchromatin segregate into different nuclear compartments, for example via microscopy approaches, a general finding that was re-emphasized by chromosome contact mapping [25,26]. Some of the heterochromatic compartments may be in a different biophysical state than euchromatin, i.e. in a phase condensate [27,28] (see below). Whether or not such condensates represent a requirement for heterochromatin spreading, for example by providing a more permissive environment, remains less understood. However, newer theoretical efforts by the Mirny and Jost/Vaillant groups have emphasized the requirement for self-attraction in three-dimensional space and compaction of the repressed domain in space for accurate reformation by spreading. In this way, modified nucleosomes are brought into close proximity with those yet to be modified [29,30]. On the more local scale, three-dimensional contacts appear necessary for efficient spreading [31,32]. Together these theoretical advances may provide a rationale for *trans*-acting factors promoting local looping, long-range contacts, spatial and/or biophysical segregation to enable heterochromatin spreading.

## 3. Factors promoting the spreading of H3K9me3 via Suv39 ‘writers’

### 3.1. Position effect variegation and the early identification of *trans*-acting regulators

A great deal of our understanding of heterochromatin formation in the last few decades derives from studies with *Drosophila* and the position-effect variegation (PEV) phenomenon. PEV occurs when a normally expressed gene becomes silenced in some cells. Silencing results from a change in the gene position, for example due to recombination (i.e. when the gene becomes juxtaposed to heterochromatin), hence the name 'position effect' [8,33–35]. Subsequently, PEV has been observed in a variety of organisms including yeasts and mammals [36,37]; but it primarily has been used in *Drosophila* as a tool to study heterochromatin formation [38,39]. A fly line with a PEV phenotype was used to screen for mutations that are either suppressors or enhancers of the phenotype. Approximately 150 genetic loci have been identified from such screens including suppressors of variegation Su(var) as well as enhancers of variegation E(var) [8,38], with a smaller fraction cloned and described. The screens revealed that the *su(var)3–9* mutant has a dominant effect over the majority of PEV modifier mutations. Later, mammalian SU(VAR)3–9 homologous (human SUV39H1 and murine Suv39h1) were shown to be histone methyltransferases (HMTs) that selectively methylate lysine 9 of the histone 3 (H3K9me) tail through their SET domains [40]. The fact that the mutant identifier, deriving from the chromosome number and linkage group, ended up matching the lysine target was a happy coincidence.

### 3.2. The central role of HP1 in positive feedback

Another important modifier identified from the screens is SU(VAR)2–5, which encodes a heterochromatin-associated protein (now called HP1a) [41,42]. HP1a interacts with many other chromosomal proteins and contains two conserved domains, an amino-terminal chromo (CD) and a carboxy-terminal chromo-shadow domain (CSD) along with a variable hinge region. HP1a belongs to a highly conserved family of chromatin proteins, with homologous present from fission yeast (Swi6, Chp2) to humans (HP1α-γ) [2,43]. The CD of HP1 binds the product of SU(VAR)3–9, H3K9me. The combination of H3K9me recognition and HP1's ability to dimerize (via the CSD) and multimerize (via the CD) makes HP1 a central spreading regulator [44–46]. HP1 binds to both the H3K9me mark on one nucleosome and the neighbouring nucleosome via a bridging interaction [47]. This nucleosome-bridging by HP1 in turn promotes H3K9me spreading [48,49]. It does so primarily via the recruitment of SU(VAR)3–9, which in turn produces more H3K9me. In *Drosophila* for instance, the N-terminus of SU(VAR)3–9 was found to interact with the HP1 CSD both *in vitro* and *in vivo* [50]. HP1 thus produces a positive feedback loop of H3K9 methylation across the chromatin fibre, bringing in more SU(VAR)3–9 at the edge of the spreading heterochromatin domain. In addition, oligomerization and bridging itself are central to the spreading process, stabilizing the nascent heterochromatin domain [2,9,47]. HP1s role in spreading has been studied in quite some detail in *S. pombe*: The cryptic loci regulator 4 (Clr4, the fission yeast SU(VAR)3–9 homologue), initiates H3K9 methylation independently of Swi6 (the main *S. pombe* HP1 homologue), but then the subsequent spreading of H3K9 methylation across the domain is Swi6-dependent [51]. Whether this occurs in mammals primarily via direct Suv39 recruitment, nucleosome bridging, or downstream interactions with other proteins (see below) is not fully clear.

### 3.3. Recruitment of additional spreading regulators by HP1

Beyond this central positive feedback and signal amplification role of HP1 it further contributes to spreading in two ways: first, HP1 recruits to H3K9me marked chromatin a diverse set of factors (more than 100 putative interacting proteins were identified by mass spectrometry) including chromatin remodellers and modifiers, such as histone deacetylases (HDACs) [52–57]. These factors, besides executing the actual gene silencing actions, produce an environment more favourable to H3K9 methylation, via removal of antagonistic activities, such as acetylation and transcription, or direct promotion of the stability of the heterochromatic state. For instance, Swi6 recruits Clr3, a fission yeast homologue of mammalian class II HDACs, which promotes spreading and maintains heterochromatin through the stabilization of H3K9me3 [58–60]. The trimethylated state is required for the transcriptionally silent heterochromatin in *S. pombe,* but also for feedback by Clr4 itself, as the CD of Clr4 is quite specific for H3K9me3 [9,16,61]*.* The HDAC function of Clr3 is also important for preventing histone modifications associated with active transcription and limiting RNA polymerase II accessibility at the repressed site (transcriptional gene silencing) [62,63]. As another example, Swi6 attracts the chaperone FACT, which is required for spreading in constitutive heterochromatin, probably via nucleosome stabilization [64–66].

### 3.4. HP1 as a regulator of heterochromatin position and biophysical state

Second, HP1 may be required to promote an environment inside the nucleus conducive to spreading. Swi6 connects heterochromatin to the nuclear periphery via the nuclear rim protein Amo1, which associates with Swi6-interacting FACT (see above) and RIXC complexes [64]. Localization of H3K9me heterochromatin to the periphery is commonly observed across systems, for example via the CEC-4 protein in worms and PRR14 to the nuclear lamina in mammals [67,68], and in some cases is critical for heterochromatin formation. How this environment promotes heterochromatic spreading and silencing remains mechanistically opaque; however, one mechanism is likely the concentration of pro-spreading factors into this niche. The ability to form a specialized heterochromatin compartment or biophysical environment is likely linked to HP1s ability to oligomerize, which is considered central to its potential to bridge nucleosomes in H3K9me spreading. Oligomerization also underlies HP1's propensity to undergo liquid–liquid phase separation (LLPS), a process where biomolecules separate into distinct liquid-like compartments within the cytoplasm or nucleus [27,28,69]. Phase separation is typically driven by weak and multivalent interactions between biomolecules. These interactions involve both folded regions, such as the CD or CSD of HP1 as well as intrinsically disordered regions, such as the N-terminal extension of HP1 or its hinge region. These associations can lead to phase separation in the cell into condensates, which can have apparent LLPS characteristics, though it remains notoriously difficult to test *in vivo* [70]. These condensates can sequester other proteins and RNAs, thereby regulating their availability for biological processes [71]. HP1 has been shown to undergo LLPS on its own *in vitro* upon phosphorylation [27,28] and induce it in chromatin in part via nucleosome distortions [72]. It has been proposed that this behaviour, and/or its ability to induce phase separation of chromatin, may underlie the formation of heterochromatin domains *in vivo.* Whether these condensates represent the cytologically observed dense domains of heterochromatin, where gene expression is typically repressed, is not fully clear. It is possible that in this context, this formed liquid droplet encloses heterochromatic sequences and helps to exclude the transcription machinery, triggering gene silencing by forming a ‘boundary’ that separates heterochromatin from the surrounding chromatin [73,74]. Whether HP1-induced phase separation applies to all H3K9me-marked heterochromatin territories is not known. HP1 containing chromocenters in the nucleoplasm that are made of alpha-satellite repeats show properties of phase-separated bodies [27,28]. By contrast, whether phase separation is involved in heterochromatin found at the nuclear periphery is unclear. It is possible that HP1 uses phase-separation to package and insulate distinct heterochromatin types.

### 3.5. Beyond HP1

Additional chromatin modifications regulate H3K9me spreading, for example by regulating chromatin structure. One such mark is trimethylation at lysine 20 of H4 (H4K20me3). This mark is produced from H4K20me1 by the SUV4-20H1 and H2 enzymes (reviewed in [75] and on its own, can compact chromatin fibres [76]. This by itself may support H3K9me spreading. In addition, SUV4-20H1 in a non-enzymatic role changes nucleosome structure when bound, promotes phase condensation of chromatin, and alters the HP1-formed chromatin condensates [77]. Both these activities of SUV4-20H might promote either a spreading compatible chromatin structure and/or biophysical environment. Further, it appears that ubiquitination (Ub) of H3K14 may be critically required to stimulate Suv39 enzymes *in situ* on the chromatin substrate and enable spreading. In *S. pombe*, the Clr4 complex contains an E3 ligase [78–80], which we now understand ubiquitinates H3K14 which binds to a partially conserved Ub-binding pocket in Clr4 [81]. Stimulation by H3K14-Ub appears conserved with mammalian Suv39 enzymes [81]. Separately, in a screen for spreading versus nucleation regulators, Greenstein *et al.* identified a complex of the Clr6 HDAC with the Fkh2 transcription factor as specifically required for heterochromatin spreading at constitutive sites. Fkh2 recruits Clr6 to nucleation-distal chromatin sites in such contexts [82]. This points to the fact that regions outside the active nucleation zone require additional manipulation of the chromatin substrate to make it compatible with heterochromatin assembly and H3K9 methylation.

## 4. Factors promoting the spreading of H3K27me3 by polycomb

### 4.1. PRC2 and PRC1 in H3K27me spreading

Heterochromatin marked by H3K27 methylation is critically involved in the control of animal and plant development, as was demonstrated via an elegant series of genetic studies in *Drosophila melanogaster* by Ed Lewis [83] and others. The central polycomb H3K27 methylase is PRC2, which consists of the evolutionary conserved Enhancer of zeste (Ezh) SET domain methylase, the H3K27me binding Embryonic ectoderm development (Eed), Suppressor of zeste 12 (Suz12) proteins, plus Rpab46/48. Repression by the polycomb system requires another enzymatic complex called PRC1, which catalyses H2AK119 ubiquitination [84]. In *Drosophila* and mammals, polycomb proteins are antagonized by trithorax group activator proteins, both systems establish a balance of activation and repression over the developmentally regulated loci such that only genes appropriate to the body segment are expressed [85]. In flies, it has become recognized that the PRC2 protein complex is recruited to specific polycomb response elements (PREs) [86,87], which have not been identified in this form in mammals. However, specific sequence contexts have been identified in mammals that appear to initially attract PRC2. These sequences contain CpG islands, and are unmethylated, along with other features [88]. Experiments that fully deplete the essential Eed protein and reintroduce it following the full loss of H3K27me have further solidified the location of these elements, which likely represent nucleation sites [18]. These sites also attract variant PRC1 complexes, triggering H2A ubiquitination and subsequent PRC2 recruitment [89]. Beyond these nucleation sites, other regions repressed by PRC2 are subject to H3K27me3 spreading from those nucleation sites. Elegant experiments tracking H3K27me3 domains in the cell cycle also indicate a continuous need for PRC2-nucleation, and later, Eed-dependent spreading, for domain reformation [90].

### 4.2. H3k27me2 versus me3 spreading

Spreading appears to be divided into short and more longer-range spreading, and here, there is a critical distinction concerning the methylation state: At first, the spreading of H3K27me2 appears relatively wide-ranging from the nucleation centre [18,91], yet the functionally critical H3K27me3 is initially more restricted [18]. Allosteric activation of Ezh2 via the Eed subunit binding to H3K27me3 [11,92], is likely required to enable further spreading of trimethylation. PRC2's activity in trimethylating H3K27 is also strongly regulated by activating PRC2 auto-methylation [93]. Oncogenic PRC2 antagonists, like H3K27M and Ezhip [94], which traps allosterically activated PRC2, instead abrogate spreading. Insertion of a regulatory step between me2 and me3 is reminiscent of Suv39 enzymes discussed above and appears to be a key gate in regulating spreading of the repressive state.

### 4.3. Regulation of H3K27me3 spreading by the chromatin substrate

Tied into this regulation of the methylation state is the regulation of these transitions by the chromatin substrate itself. Beyond the influence of histone modifications other than H3K27, which are not further discussed here, a central concept already touched on is regulation of nucleosome density and stability. In the case of PRC2, it was shown that the nucleosome spacing influences nucleosome methylation activity by PRC2, with 40 bp being the ideal spacing for methylation on dinucleosomes and arrays [95]. This is consistent with the results from structural biology that show PRC2 directly reaching from a methylated to a unmethylated substrate nucleosome with 30–35 bp spacing [96]. Another report showed that a high density of nucleosome *in cis* is required for stimulation [97]. Recent evidence indicates one key factor that regulates the optimal nucleosome arrangement for spreading may be the linker histone H1. *in vitro* and *in vivo* evidence [98] seems to support a role for H1 creating a chromatin structure that stimulates spreading by PRC2. The details of the precise nucleosome arrangement produced by H1 and favoured by PRC2, and how it relates to prior work on nucleosome spacing and density remains to be determined.

### 4.4. Trans-H3K27me3 spreading

As alluded to above, H3K27me3 after this initial local spreading is capable of long-range spreading apparently both *in cis* and *in trans.* How is this long-range spreading facilitated? The answer may reside in the biophysical nature of the polycomb domains, which appear to form ‘polycomb bodies’ (PBs) in the nucleus [99]. There, PBs bring together relatively distal sequences in a manner that is not only independent of the general looping and architecture regulatory pair CTCF and cohesion, but rather appears to be antagonized by it [100,101]. Nucleation sites appear distally contacted through canonical PRC1 complexes. These canonical PRC1 complexes, unlike its variant PRC1 complex cousins, are less capable of the central enzymatic activity of PRC1, H2A ubiquitination, but instead are more prone to oligomerize [89,102–106]. Such capacity to oligomerize has long been associated with PRC1 proteins such as the *Drosophila* Ph [107] as well as with the mammalian homologue Phc1/2 [103]. Similarly, the *Drosophila* PSC oligomerizes [108] and the *Drosophila* protein has been shown to mediate nucleosome compaction [102]. Further, hetero-oligomerization between the PRC1 component is thought to be essential in phase separation and likely PB formation [109–111]. It is likely that the Phc1 and the PSC homologue Bmi-1 (PCGF4) in canonical PRC1, in addition to CBX2, could mediate clustering and phase separation [106,109,111]. These clustered nucleation sites then allow PRC2 to spread H3K27me3 across distal regions, enabling PRC2 to exit the local nucleation environment [18], a model that is also consistent with theoretical modelling of heterochromatin spreading in three dimensions via self-attraction [29,30]. Such nucleation site clustering, presumably in PBs, also apparently ensures redundancy in targeting spreading sites from several spatially adjacent nucleation sites, which may be at various distances in genomic space. To what degree the biophysical environment inside the PBs is important for the spreading process itself remains to be determined.

### 4.5. Potential roles of PRC2 oligomerization

While PRC1 oligomerization appears to allow the connection of distal sites, this type of oligomer formation is different from the coupling of oligomerization to writer product reading we saw for the Suv39 system. There is no apparent evidence that PRC1 oligomerization is connected to H3K27me reading. However, recent evidence implies that different PRC2 types can form dimeric complexes. For example, EZH1 containing PRC2 can dimerize on the nucleosome [112]. The structure of that complex, with two ‘reading’ Eed domains facing outward, may directly couple ‘writing’ to cross-nucleosome spreading. How this is related to structures of EZH2 PRC2 [96,113,114] and when dimerization is active *in vivo* remains to be determined. Finally, whether a linked oligomerization/product recognition cycle is operational for this system is not clear.

### 4.6. Lessons from plants: coupling nucleation and spreading

Interestingly, in plants, H3K27me3 spreading shares some features with Suv39 H3K9me3 spreading, as uniquely in plants, an HP1 protein LIKE HETEROCHROMATIN PROTEIN 1 (LHP1) appears to act downstream or in parallel to PRC2 [115]. LHP1 binds H3K27me3 [116,117] that is deposited by PRC2 over flowering time loci and appears to aid in the spreading of this mark [118,119]. Intriguingly, spreading of the mark beyond nucleation sites is required for the epigenetic stability of the domain [119], highlighting the unique role of spreading in intergenerational maintenance.

## 5. Spreading by SETDB1

### 5.1. KAP1, KRAB-zfps and SETDB1s initial recruitment

SET domain bifurcated 1 (SETDB1) is a specific H3K9 methyltransferase that primarily acts in euchromatin sequences to silence retroelements or developmental regulators. An extensive amount of research has been conducted on the recruitment of SETDB1 to retroelements. Nucleation requires recognition of target sites by KRAB zinc finger proteins (KRAB-zfps), which then recruit SETDB1 via the critical co-repressor KAP1 (also known as TRIM28) [120,121]. KAP1 forms a complex with SETDB1 [122], which requires the intramolecular SUMOylation of KAP1 [123–125]. When KAP1 is depleted, SETDB1 enrichment in class I/II ERVs is decreased [120], indicating that the formation of the KAP-KRAB-zfps complex is a central step in SETDB1 recruitment to ERVs.

### 5.2. Pathways of H3K9me3 spreading by SETDB1

A first indication of H3K9me3 spreading by SETDB1 is that KRAB/KAP1 binding sites may be found only in one region of the retroelement, while H3K9me3 enrichment occurs throughout the entire retroelement [126,127]. Separately, SETDB1-generated H3K9me3 can repress the transcription of genes located distally to KRAB-zfp binding sites, in some cases up to tens of kilobases away [128–131]. This H3K9me3 spreading via SETDB1 may be facilitated by product recognition feedback. There are two potential pathways for this, outside the SETDB1 enzyme subunit, which remain underexplored:

1. HP1 may perform ‘double duty’ as a key spreading regulator for both Suv39 and SETDB1. Targeting of HP1*α*, HP1*β* and HP1*γ* to heterologous loci is sufficient to induce the recruitment of SETDB1 and deposition of H3K9me3 [132,133]. In mESCs, ERVs are enriched in HP1*α*, HP1*β* and HP1*γ* and this occurs in part due to SETDB1-deposited H3K9me3 [120]. There is a slight derepression in the expression of ERVs, as well as a partial reduction in H3K9me3 around the ERVs in HP1*β* KO mESCs [133,134]. The HP1 protein is therefore thought to be partially implicated in SETDB1-mediated H3K9me3 spreading.
2. Beyond HP1, a specialized complex has been identified that may be required for SETDB1 to mark target domains with H3K9me3. Using a similar strategy to find the PEV elements in drosophila cells, a non-lethal forward genetic screen conducted in haploid human KBM7 cells identified the human silencing hub (HUSH), which consists of the proteins TASOR, MPP8 and periphilin [130]. TASOR appears to be the hub of HUSH, providing a platform for assembling the other subunits, and has been identified as a pseudo-Poly (ADP-ribose) polymerase essential for HUSH complex assembly [135]. One central activity of HUSH is the recruitment of both SETDB1, as well as another factor MORC2 to sites of initial H3K9me3 in retroelements. MORC2 appears to have ATP-dependent remodelling activity key to the compaction of the underlying chromatin [130,136,137]. Whether HUSH is directly involved in H3K9me3 spreading is still contentious. The mechanisms by which HUSH may be involved in spreading comes back to product recognition. The CD of MPP8 binds H3K9me2 and H3K9me3 [138]. Hence MPP8 could ‘read’ the product of SETBD1 on nucleosomes. However, MPP8 also recognizes the methylation of an H3-like mimic sequence found in other proteins, such as ATF7IP, the nuclear chaperone of SETDB1 [139]. This methylation on ATF7IP is thought to be partially required for HUSH-dependent silencing [140]. Another critical piece of information is that the CD of MPP8 can form dimers [141]. Since ATF7IP is required for SETDB1 stability [139], a model could be envisioned where following recruitment of SETDB1 to a transgene, initial methylation occurs on H3K9 and AT7IP. MPP8 dimers could bridge chromatin and ATF7IP, recruiting the active form of SETDB1, which would constitute a read-write cycle for outward spreading over HUSH targets. This would nicely mirror parts of the Suv39 model where stabilization of the H3K9me substrate, multimerization and writer recruitment enable spreading. Yet, a H3K9me3 read-write mechanism involving MPP8 alone and ATF7IP/SETDB1 is probably too simplistic: HUSH-dependent lentiviral reporter repression requires both TASOR and Periphilin, and the MPP8 chromodomain is not required for the maintenance of repression [130]. However, the establishment of repression, so possibly the initial domain specification, is dependent on the MPP8 chromo domain [130].

It is also noteworthy that HUSH targets are enriched within transcriptionally active chromatin, as opposed to classical heterochromatin regulators [142,143], which is akin to de novo spreading, for example, by the polycomb system in cell fate decisions. Together spreading by the SETDB1 system is still a new field, especially with the relatively recent discovery of HUSH. Whether it follows a ‘read–write’ type of mechanism and to what degree this interfaces with HP1 remains to be determined.

## 6. Spreading via SIR proteins

### 6.1. The SIR2/3/4 system

Yeast *Saccharomyces cerevisiae* SIR genes encode a family of nuclear proteins that are targeted to specific genomic sequences and targeted for silencing. SIR proteins are associated with three classes of genomic sequences: subtelomeres (which serve as the principal storage sites for the SIR proteins), silenced mating-type loci (i.e. HMR and HML) and rDNA sequences. To efficiently silence the HM loci, Sir1, Sir2, Sir3 and Sir4 are required, while Sir2, Sir3 and Sir4 are required to silence subtelomeres effectively. The central writer of the SIR complement is Sir2 which is a NAD-dependent deacetylase that targets histone H3 and H4.

Silent chromatin assembles in two stages at the molecular level: nucleation and spreading. Nucleation occurs when the Sir2/3/4 complex is recruited to silencers for the first time. The spreading step occurs following the assembling of the extended domain of silent chromatin by the complex. As a result of the intrinsic properties of the SIR proteins, the nucleation and spreading steps are closely linked. Nucleation without spreading and spreading without nucleation can be studied through mutations and other experimental manipulations (e.g. [144]).

The process of nucleation relatively well understood. The Sir2/3/4 complex is recruited to proteins bound at silencers by a network of interactions. Both Sir3 and Sir4 associate with the transcription factor Repressor-activator protein 1 (Rap1), while Sir4 also associates with Origin Recognition Complex (ORC)-bound Sir1 [145,146]. The transcription factor ARS-binding factor (Abf1) also cooperates in this process at HMR and HML [147]. Mutants of Sir2 that exhibit a catalytic defect restrict the Sir2/3/4 complex to silencers [148–150]. This suggests that the deacetylation of histones by Sir2 triggers the transition from nucleation to spreading.

### 6.2. SIR spreading: coupling of deacetylation and Sir3 oligomeric engagement

According to the original sequential model of spreading, Sir2 first deacetylates the nucleosomes adjacent to silencers, creating additional recruitment sites for Sir2/3/4 complexes [151]. Sir3 prefers to bind to deacetylated H4 tails, specifically amino acid H4K16 [6,152]. In an alternative view, based on the observed affinity of the Sir2/3/4 complex for acetylated H4K16, the complex is thought to bind acetylated nucleosomes first, and then acquire additional stability via deacetylation of H4K16 and docking of Sir3 to the deacetylated tails [153]. As a result of successive spreading, Sir2/3/4 binding, histone deacetylation and interactions between Sir2/3/4 complexes expand the silent chromatin domain until either a barrier is reached, or the pool of free SIR proteins falls below a threshold that facilitates efficient binding. In this view, the sequential spreading of Sir2/3/4 complexes is analogous to a linear polymerization reaction.

A more detailed mechanism has been proposed recently, involving Sir3 and Sir4 propagation along the fibre. The domain architecture of Sir3 has some conceptual similarities to HP1 in that it contains a nucleosome binding and dimerization domain. The N-terminus of Sir3 contains a bromo-adjacent homology (BAH) domain (amino acids 11–196). Many chromatin-associated proteins, including Orc1, Dnmt1, Rsc1, Rsc2 and Mta1 [154,155], contain the BAH domain, which is involved in nucleosome binding [156,157], and in Sir3's case specifically, nucleosomes that are the products of Sir2 (see below). Separately, Sir3's winged helix-turn-helix domain mediates self-interaction [158] and dimerization. This dimer/multimerization of Sir3 is likely at the heart of spreading. A model driven by biophysical measurements proposes that Sir3 spreads along the chromatin fibre using an ‘interrupted bridges’ [159] mechanism, where a Sir3 dimer bridges from one face of the nucleosome to the adjacent nucleosome face (figure 2). Further functional crosstalk with the enzymatic step via Sir2 also may feed directly into the oligomerization process.

### 6.3. Possible modulation by the Sir2 deacetylation by-product O-acetyl-ADP-ribose

But unlike the case of HP1, there is another interesting wrinkle to the SIR system, and that is regulation by one of the reaction products: Sir2 and other NAD-dependent protein Sir2/Class III HDAC uses NAD as a cofactor for deacetylation. Two products derive from NAD: nicotinamide and O-acetyl-ADP-ribose (AAR) [160,161]. AAR is associated with silent heterochromatin domains and demonstrates a similar pattern to that of Sir2 [162]. Intriguingly, the *in vitro* association of SIR2-3-4 complex and Sir3 alone with recombinant trinucleosomes is enhanced by AAR [163]. Similar modulation of binding to purified yeast nucleosomes was also reported [157,164]. The effect of AAR on chromatin epigenetic gene silencing has been demonstrated *in vivo* [165]. Moreover, previous observations regarding the role of AAR in the assembly of the SIR complex, as discussed above, strongly suggest that AAR binds to at least one of the SIR proteins [152]. For example, AAR might associate with the AAA ATPase-like domain within SIR3's C-terminus [158,166,167]. However, there is no strong direct evidence that AAR binds Sir3, instead, evidence supports binding to Sir2 [162], leaving the mechanism of action unclear. Even if AAR enhances the efficiency of Sir3-nucleosome complex formation, it does not appear that AAR is necessary for SIR silencing. This is because silent chromatin can be assembled *in vivo* using Hos3 (where Hos3 is targeted by a SIR3-Hos3 fusion), a deacetylase of the Rpd3 family that does not consume NAD nor produce AAR [168]. Despite the absence of all NAD-dependent deacetylases, the chimera produced robust transcriptional silencing. Therefore, if AAR is involved in the spreading of silencing, it may act to modulate, rather than drive the process.

### 6.4. Antagonism to SIR spreading

Spreading via Sir3 is also downregulated or limited in several ways. A prominent example is the acetylation of H4K16, which has been shown to impact Sir3 chromatin association by mutational analyses, ChIP and co-immunoprecipitation studies, as well as biochemical studies [149–151,153,157,169–171]. The co-crystal structure of the nucleosome and the Sir3 BAH domain [10] visualized how this antagonism by H4K16 acetylation, but also H3K79 methylation would regulate Sir3 association with the nucleosome surface. A majority of the BAH domain's electrostatic contacts are with histone residues K16 and H18 in the H4 N terminal tail. A significant decrease in the affinity of Sir3 for the nucleosome is expected to occur as a result of the acetylation of K16. This is consistent with previous studies, which have indicated that acetylation has a 1000-fold impact [157]. Thus, Sir3/4 spreading, like that of HP1, requires recognition of the writer enzyme product on chromatin, in this case, a deacetylated H4 tail.

## 7. Conclusion

In the above, we have attempted to summarize what is known about how *trans-*acting factors regulate of heterochromatin spreading by writers in four systems. Some common principles emerge about how *trans*-acting factors promote the spreading of heterochromatin by writer enzymes. These are also summarized in figure 2.

First, *trans*-acting factors directly promote the positive feedback inherent in most heterochromatic writers. They do so by acting as a second, redundant feedback layer. The redundant layer, obvious in Suv39, SIR, and likely SETDB1 systems, consists of ‘reader’ proteins binding the writer product on chromatin and recruiting more writers via direct physical interactions.

Second, through processes of oligomerization on the chromatin substrate, writers like HP1 and Sir3 stabilize the structure and ensure that opportunities for redundant feedback are present across the forming heterochromatin domain. How the writers however do not end up getting ‘trapped’ in the core of the domain remains unresolved. A hint at a possible mechanism is differential preferences for methylation states of the chromatin mark in systems where methylation is the instructive chemical change (hence, not in the case of Sir2). Swi6 and Clr4 have differential preferences for H3K9me2 and me3, with Clr4 strongly preferring H3K9me3 [9,61]. Since H3K9me2 is more abundant than H3K9me3, one can consider one mark the assembly and the other the spreading and silencing mark [16].

Third, *trans*-acting factors likely promote spreading by shaping a microenvironment conducive to spreading, in part via nucleosome stabilization and possibly by producing an altered biophysical environment. Spatial segregation and self-attraction of heterochromatin has been predicted to be required for efficient spreading and inheritance by modelling approaches [29,30]. HP1 appears to be involved in anchoring some but not all H3K9me domains to the nuclear periphery, for example, those that fall into lamin-associated domains (controlled largely by G9a/GLP which we did not discuss in this review). The nuclear periphery in the case of fission yeast may be enriched for factors that stabilize nucleosomes [64]. Nucleosome stabilization, in turn, is central for efficient spreading, and especially for production of the trimethylated state in the case of H3K9me and K27me systems, which drives positive feedback. This state is also favoured by deacetylases that are recruited to the spreading zone. Finally, at least HP1 and PRC1 appear to be involved in forming condensates or condensate-like domains *in vivo* that may be required to promote the stability of heterochromatin and its spreading. In principle, condensate formation may promote writer enzyme activity directly, for example by increasing local concentration and altering the chromatin structure, or by rejecting antagonistic factors, such as excluding acetylases, transcription factors, or RNA polymerase (figure 2). In either case, resolving how condensates influence heterochromatin spreading remains a very active area of research.

However, there are also unique aspects not shared across the different heterochromatin systems. Firstly, the polycomb system's ability to perform long-distance spreading via PRC1 clusters in PBs does not have an exact parallel for other systems, and may be required for the reliable silencing of large developmental loci via redundant of spreading from dispersed nucleation sites [18]. SIR proteins may be regulated in their chromatin interactions via the Sir2 writer NAD deacetylation reaction by-product AAR, achieving a potential level of feedback lacking in the S-adenosyl methionine-dependent methyltransferase writers Suv39, PRC2 and SETDB1. The by-product in this case, S-adenosyl homocysteine is largely inhibitory via product inhibition. The interactions of HP1/Swi6 appear particularly diverse and contain inbuilt autoregulation not observed to the same extent elsewhere. In a facet not reviewed above, HP1/Swi6 even directly recruits negative heterochromatin spreading regulators such as Epe1 [172,173] in a manner tightly coordinated with H3K9 methylation. This restrains propagation of heterochromatin beyond the natural borders in fission yeast.

More work remains on unravelling how some of these unique mechanisms shape the spreading reaction and how they interface with the common operating principles discussed above.

## Data Availability

This article has no additional data.

## References

[1] Heitz E. 1928 Das Heterochromatin der Moose. Jahrb Wiss Bot. **69**, 762-818.

[2] Grewal SIS, Jia S. 2007 Heterochromatin revisited. Nat. Rev. Genet. **8**, 35-46. (10.1038/nrg2008) 17173056

[3] Moazed D. 2009 Small RNAs in transcriptional gene silencing and genome defence. Nature **457**, 413-420. (10.1038/nature07756) 19158787 PMC3246369

[4] Talbert PB, Henikoff S. 2006 Spreading of silent chromatin: inaction at a distance. Nat. Rev. Genet. **7**, 793-803. (10.1038/nrg1920) 16983375

[5] Hecht A, Strahl-Bolsinger S, Grunstein M. 1996 Spreading of transcriptional repressor SIR3 from telomeric heterochromatin. Nature **383**, 92-96. (10.1038/383092a0) 8779721

[6] Hecht A, Laroche T, Strahl-Bolsinger S, Gasser SM, Grunstein M. 1995 Histone H3 and H4 N-termini interact with SIR3 and SIR4 proteins: a molecular model for the formation of heterochromatin in yeast. Cell **80**, 583-592. (10.1016/0092-8674(95)90512-X) 7867066

[7] Renauld H, Aparicio OM, Zierath PD, Billington BL, Chhablani SK, Gottschling DE. 1993 Silent domains are assembled continuously from the telomere and are defined by promoter distance and strength, and by SIR3 dosage. Genes Dev. **7**, 1133-1145. (10.1101/gad.7.7a.1133) 8319906

[8] Elgin SC, Reuter G. 2013 Position-effect variegation, heterochromatin formation, and gene silencing in Drosophila. Cold Spring Harb. Perspect. Biol. **5**, a017780. (10.1101/cshperspect.a017780) 23906716 PMC3721279

[9] Al-Sady B, Madhani HD, Narlikar GJ. 2013 Division of labor between the chromodomains of HP1 and Suv39 methylase enables coordination of heterochromatin spread. Mol. Cell **51**, 80-91. (10.1016/j.molcel.2013.06.013) 23849629 PMC3752401

[10] Armache KJ, Garlick JD, Canzio D, Narlikar GJ, Kingston RE. 2011 Structural basis of silencing: Sir3 BAH domain in complex with a nucleosome at 3.0 A resolution. Science **334**, 977-982. (10.1126/science.1210915) 22096199 PMC4098850

[11] Margueron R et al. 2009 Role of the polycomb protein EED in the propagation of repressive histone marks. Nature **461**, 762-767. (10.1038/nature08398) 19767730 PMC3772642

[12] Muller MM, Fierz B, Bittova L, Liszczak G, Muir TW. 2016 A two-state activation mechanism controls the histone methyltransferase Suv39h1. Nat. Chem. Biol. **12**, 188-193. (10.1038/nchembio.2008) 26807716 PMC4876634

[13] Zhang K, Mosch K, Fischle W, Grewal SIS. 2008 Roles of the Clr4 methyltransferase complex in nucleation, spreading and maintenance of heterochromatin. Nat. Struct. Mol. Biol. **15**, 381-388. (10.1038/nsmb.1406) 18345014

[14] Dodd IB, Micheelsen MA, Sneppen K, Thon G. 2007 Theoretical analysis of epigenetic cell memory by nucleosome modification. Cell **129**, 813-822. (10.1016/j.cell.2007.02.053) 17512413

[15] Garcia JF, Dumesic PA, Hartley PD, El-Samad H, Madhani HD. 2010 Combinatorial, site-specific requirement for heterochromatic silencing factors in the elimination of nucleosome-free regions. Genes Dev. **24**, 1758-1771. (10.1101/gad.1946410) 20675407 PMC2922504

[16] Jih G, Iglesias N, Currie MA, Bhanu NV, Paulo JA, Gygi SP, Garcia BA, Moazed D. 2017 Unique roles for histone H3K9me states in RNAi and heritable silencing of transcription. Nature **547**, 463-467 (10.1038/nature23267) 28682306 PMC5576860

[17] Chin HG, Patnaik D, Estève P-O, Jacobsen SE, Pradhan S. 2006 Catalytic properties and kinetic mechanism of human recombinant Lys-9 histone H3 methyltransferase SUV39H1: participation of the chromodomain in enzymatic catalysis. Biochemistry **45**, 3272-3284. (10.1021/bi051997r) 16519522

[18] Oksuz O et al. 2018 Capturing the onset of PRC2-mediated repressive domain formation. Mol. Cell **70**, 1149-1162. (10.1016/j.molcel.2018.05.023) 29932905 PMC7700016

[19] Zee BM, Britton L-MP, Wolle D, Haberman DM, Garcia BA. 2012 Origins and formation of histone methylation across the human cell cycle. Mol. Cell. Biol. **32**, 2503-2514. (10.1128/MCB.06673-11) 22547680 PMC3434498

[20] Binda O, Leroy G, Bua DJ, Garcia BA, Gozani O, Richard S. 2010 Trimethylation of histone H3 lysine 4 impairs methylation of histone H3 lysine 9: regulation of lysine methyltransferases by physical interaction with their substrates. Epigenetics **5**, 767-775. (10.4161/epi.5.8.13278) 21124070 PMC3052887

[21] Lechner CC, Agashe ND, Fierz B. 2016 Traceless synthesis of asymmetrically modified bivalent nucleosomes. Angew. Chem. Int. Ed Engl. **55**, 2903-2906. (10.1002/anie.201510996) 26806951

[22] Schmitges FW et al. 2011 Histone methylation by PRC2 is inhibited by active chromatin marks. Mol. Cell **42**, 330-341. (10.1016/j.molcel.2011.03.025) 21549310

[23] Altaf M, Utley RT, Lacoste N, Tan S, Briggs SD, Côté J. 2007 Interplay of chromatin modifiers on a short basic patch of histone H4 tail defines the boundary of telomeric heterochromatin. Mol. Cell **28**, 1002-1014. (10.1016/j.molcel.2007.12.002) 18158898 PMC2610362

[24] Carmen AA, Milne L, Grunstein M. 2002 Acetylation of the yeast histone H4 N terminus regulates its binding to heterochromatin protein SIR3. J. Biol. Chem. **277**, 4778-4781. (10.1074/jbc.M110532200) 11714726

[25] Falk M et al. 2019 Heterochromatin drives compartmentalization of inverted and conventional nuclei. Nature **570**, 395-399. (10.1038/s41586-019-1275-3) 31168090 PMC7206897

[26] Lieberman-Aiden E et al. 2009 Comprehensive mapping of long-range interactions reveals folding principles of the human genome. Science **326**, 289-293. (10.1126/science.1181369) 19815776 PMC2858594

[27] Larson AG, Elnatan D, Keenen MM, Trnka MJ, Johnston JB, Burlingame AL, Agard DA, Redding S, Narlikar GJ. 2017 Liquid droplet formation by HP1*α* suggests a role for phase separation in heterochromatin. Nature **547**, 236-240. (10.1038/nature22822) 28636604 PMC5606208

[28] Strom AR, Emelyanov AV, Mir M, Fyodorov DV, Darzacq X, Karpen GH. 2017 Phase separation drives heterochromatin domain formation. Nature **547**, 241-245. (10.1038/nature22989) 28636597 PMC6022742

[29] Abdulla AZ, Vaillant C, Jost D. 2022 Painters in chromatin: a unified quantitative framework to systematically characterize epigenome regulation and memory. Nucleic Acids Res. **50**, 9083-9104. (10.1093/nar/gkac702) 36018799 PMC9458448

[30] Owen JA, Osmanović D, Mirny LA. 2022 Design principles of 3D epigenetic memory systems. 10.1101/2022.09.24.509332

[31] Erdel F, Greene EC. 2016 Generalized nucleation and looping model for epigenetic memory of histone modifications. Proc. Natl Acad. Sci. USA **113**, E4180-9. (10.1073/pnas.1605862113) 27382173 PMC4961120

[32] Katava M, Shi G, Thirumalai D. 2022 Chromatin dynamics controls epigenetic domain formation. Biophys. J. **121**, 2895-2905. (10.1016/j.bpj.2022.07.001) 35799447 PMC9388564

[33] Girton JR, Johansen KM. 2008 Chapter 1 chromatin structure and the regulation of gene expression: the lessons of PEV in *Drosophila*. In Advances in genetics, pp. 1-43. Elsevier. 10.1016/S0065-2660(07)00001-6 18282501

[34] Schotta G, Ebert A, Dorn R, Reuter G. 2003 Position-effect variegation and the genetic dissection of chromatin regulation in *Drosophila*. Semin. Cell Dev. Biol. **14**, 67-75. (10.1016/S1084-9521(02)00138-6) 12524009

[35] Schulze SR, Wallrath LL. 2007 Gene regulation by chromatin structure: paradigms established in Drosophila melanogaster. Annu. Rev. Entomol. **52**, 171-192. (10.1146/annurev.ento.51.110104.151007) 16881818

[36] Allshire RC, Ekwall K. 2015 Epigenetic regulation of chromatin states in *Schizosaccharomyces pombe*. Cold Spring Harb. Perspect. Biol. **7**, a018770. (10.1101/cshperspect.a018770) 26134317 PMC4484966

[37] Brockdorff N, Turner BM. 2015 Dosage compensation in mammals. Cold Spring Harb. Perspect. Biol. **7**, a019406. (10.1101/cshperspect.a019406) 25731764 PMC4355265

[38] Grigliatti T. 1991 Position-effect variegation–an assay for nonhistone chromosomal proteins and chromatin assembly and modifying factors. Methods Cell Biol. **35**, 587-627. (10.1016/S0091-679X(08)60588-9) 1685760

[39] Reute G, Spierer P. 1992 Position effect variegation and chromatin proteins. BioEssays **14**, 605-612. (10.1002/bies.950140907) 1365916

[40] Rea S et al. 2000 Regulation of chromatin structure by site-specific histone H3 methyltransferases. Nature **406**, 593-599. (10.1038/35020506) 10949293

[41] Eissenberg JC, James TC, Foster-Hartnett DM, Hartnett T, Ngan V, Elgin SC. 1990 Mutation in a heterochromatin-specific chromosomal protein is associated with suppression of position-effect variegation in Drosophila melanogaster. Proc. Natl Acad. Sci. USA **87**, 9923-9927. (10.1073/pnas.87.24.9923) 2124708 PMC55286

[42] James TC, Elgin SCR. 1986 Identification of a nonhistone chromosomal protein associated with heterochromatin in *Drosophila melanogaster* and its gene. Mol. Cell. Biol. **6**, 3862-3872. (10.1128/mcb.6.11.3862-3872.1986) 3099166 PMC367149

[43] Huisinga KL, Brower-Toland B, Elgin SCR. 2006 The contradictory definitions of heterochromatin: transcription and silencing. Chromosoma **115**, 110-122. (10.1007/s00412-006-0052-x) 16506022

[44] Bannister AJ, Zegerman P, Partridge JF, Miska EA, Thomas JO, Allshire RC, Kouzarides T. 2001 Selective recognition of methylated lysine 9 on histone H3 by the HP1 chromo domain. Nature **410**, 120-124. (10.1038/35065138) 11242054

[45] Cowieson NP, Partridge JF, Allshire RC, Mclaughlin PJ. 2000 Dimerisation of a chromo shadow domain and distinctions from the chromodomain as revealed by structural analysis. Curr. Biol. **10**, 517-525. (10.1016/S0960-9822(00)00467-X) 10801440

[46] Lachner M, O'carroll N, Rea S, Mechtler K, Jenuwein T. 2001 Methylation of histone H3 lysine 9 creates a binding site for HP1 proteins. Nature **410**, 116-120. (10.1038/35065132) 11242053

[47] Canzio D, Chang EY, Shankar S, Kuchenbecker KM, Simon MD, Madhani HD, Narlikar GJ, Al-Sady B. 2011 Chromodomain-mediated oligomerization of HP1 suggests a nucleosome-bridging mechanism for heterochromatin assembly. Mol. Cell **41**, 67-81. (10.1016/j.molcel.2010.12.016) 21211724 PMC3752404

[48] Hiragami-Hamada K et al. 2016 Dynamic and flexible H3K9me3 bridging via HP1*β* dimerization establishes a plastic state of condensed chromatin. Nat. Commun. **7**, 11310. (10.1038/ncomms11310) 27090491 PMC4838890

[49] Machida S, Takizawa Y, Ishimaru M, Sugita Y, Sekine S, Nakayama J, Wolf M, Kurumizaka H. 2018 Structural basis of heterochromatin formation by human HP1. Mol. Cell **69**, 385-397. (10.1016/j.molcel.2017.12.011) 29336876

[50] Schotta G. 2002 Central role of *Drosophila* SU(VAR)3-9 in histone H3-K9 methylation and heterochromatic gene silencing. Embo. J. **21**, 1121-1131. (10.1093/emboj/21.5.1121) 11867540 PMC125909

[51] Hall IM, Shankaranarayana GD, Noma KI, Ayoub N, Cohen A, Grewal SIS. 2002 Establishment and maintenance of a heterochromatin domain. Science **297**, 2232-2237. (10.1126/science.1076466) 12215653

[52] Fischer T, Cui B, Dhakshnamoorthy J, Zhou M, Rubin C, Zofall M, Veenstra TD, Grewal SIS. 2009 Diverse roles of HP1 proteins in heterochromatin assembly and functions in fission yeast. Proc. Natl Acad. Sci. USA **106**, 8998-9003. (10.1073/pnas.0813063106) 19443688 PMC2690032

[53] Iglesias N et al. 2020 Native chromatin proteomics reveals a role for specific nucleoporins in heterochromatin organization and maintenance. Mol. Cell **77**, 51-66. (10.1016/j.molcel.2019.10.018) 31784357 PMC7224636

[54] Lechner MS, Schultz DC, Negorev D, Maul GG, Rauscher FJ. 2005 The mammalian heterochromatin protein 1 binds diverse nuclear proteins through a common motif that targets the chromoshadow domain. Biochem. Biophys. Res. Commun. **331**, 929-937. (10.1016/j.bbrc.2005.04.016) 15882967

[55] Motamedi MR, Hong E-JE, Li X, Gerber S, Denison C, Gygi S, Moazed D. 2008 HP1 proteins form distinct complexes and mediate heterochromatic gene silencing by nonoverlapping mechanisms. Mol. Cell **32**, 778-790. (10.1016/j.molcel.2008.10.026) 19111658 PMC2735125

[56] Smothers JF, Henikoff S. 2000 The HP1 chromo shadow domain binds a consensus peptide pentamer. Curr. Biol. **10**, 27-30. (10.1016/S0960-9822(99)00260-2) 10660299

[57] Swenson JM, Colmenares SU, Strom AR, Costes SV, Karpen GH. 2016 The composition and organization of Drosophila heterochromatin are heterogeneous and dynamic. eLife **5**, e16096. (10.7554/eLife.16096) 27514026 PMC4981497

[58] Aygun O, Mehta S, Grewal SI. 2013 HDAC-mediated suppression of histone turnover promotes epigenetic stability of heterochromatin. Nat. Struct. Mol. Biol. **20**, 547-554. (10.1038/nsmb.2565) 23604080 PMC3661211

[59] Yamada T, Fischle W, Sugiyama T, Allis CD, Grewal SIS. 2005 The nucleation and maintenance of heterochromatin by a histone deacetylase in fission yeast. Mol. Cell **20**, 173-185. (10.1016/j.molcel.2005.10.002) 16246721

[60] Zofall M, Sandhu R, Holla S, Wheeler D, Grewal SIS. 2022 Histone deacetylation primes self-propagation of heterochromatin domains to promote epigenetic inheritance. Nat. Struct. Mol. Biol. **29**, 898-909. (10.1038/s41594-022-00830-7) 36064597 PMC10357965

[61] Schalch T, Job G, Noffsinger VJ, Shanker S, Kuscu C, Joshua-Tor L, Partridge JF. 2009 High-affinity binding of Chp1 chromodomain to K9 methylated histone H3 is required to establish centromeric heterochromatin. Mol. Cell **34**, 36-46. (10.1016/j.molcel.2009.02.024) 19362535 PMC2705653

[62] Bjerling P, Silverstein RA, Thon G, Caudy A, Grewal S, Ekwall K. 2002 Functional divergence between histone deacetylases in fission yeast by distinct cellular localization and in vivo specificity. Mol. Cell. Biol. **22**, 2170-2181. (10.1128/MCB.22.7.2170-2181.2002) 11884604 PMC133699

[63] Sugiyama T, Cam HP, Sugiyama R, Noma K, Zofall M, Kobayashi R, Grewal SIS. 2007 SHREC, an effector complex for heterochromatic transcriptional silencing. Cell **128**, 491-504. (10.1016/j.cell.2006.12.035) 17289569

[64] Holla S, Dhakshnamoorthy J, Folco HD, Balachandran V, Xiao H, Sun L, Wheeler D, Zofall M, Grewal SIS. 2020 Positioning heterochromatin at the nuclear periphery suppresses histone turnover to promote epigenetic inheritance. Cell **180**, 150-164. (10.1016/j.cell.2019.12.004) 31883795 PMC7102895

[65] Lejeune E, Bortfeld M, White SA, Pidoux AL, Ekwall K, Allshire RC, Ladurner AG. 2007 The Chromatin-remodeling factor FACT contributes to centromeric heterochromatin independently of RNAi. Curr. Biol. **17**, 1219-1224. (10.1016/j.cub.2007.06.028) 17614284 PMC7612134

[66] Murawska M, Greenstein RA, Schauer T, Olsen KCF, Ng H, Ladurner AG, Al-Sady B, Braun S. 2021 The histone chaperone FACT facilitates heterochromatin spreading by regulating histone turnover and H3K9 methylation states. Cell Rep. **37**, 109944. (10.1016/j.celrep.2021.109944) 34731638 PMC8608617

[67] Gonzalez-Sandoval A et al. 2015 Perinuclear anchoring of H3K9-methylated chromatin stabilizes induced cell fate in *C. elegans* Embryos. Cell **163**, 1333-1347. (10.1016/j.cell.2015.10.066) 26607792

[68] Poleshko A, Mansfield KM, Burlingame CC, Andrake MD, Shah NR, Katz RA. 2013 The human protein PRR14 tethers heterochromatin to the nuclear lamina during interphase and mitotic exit. Cell Rep. **5**, 292-301. (10.1016/j.celrep.2013.09.024) 24209742 PMC3867587

[69] Sanulli SJ, Narlikar G. 2020 Liquid-like interactions in heterochromatin: implications for mechanism and regulation. Curr. Opin. Cell Biol. **64**, 90-96. (10.1016/j.ceb.2020.03.004) 32434105 PMC7371496

[70] Mcswiggen DT, Mir M, Darzacq X, Tjian R. 2019 Evaluating phase separation in live cells: diagnosis, caveats, and functional consequences. Genes Dev. **33**, 1619-1634. (10.1101/gad.331520.119) 31594803 PMC6942051

[71] Hyman AA, Weber CA, Jülicher F. 2014 Liquid-liquid phase separation in biology. Annu. Rev. Cell Dev. Biol. **30**, 39-58. (10.1146/annurev-cellbio-100913-013325) 25288112

[72] Sanulli S, Trnka MJ, Dharmarajan V, Tibble RW, Pascal BD, Burlingame AL, Griffin PR, Gross JD, Narlikar GJ. 2019 HP1 reshapes nucleosome core to promote heterochromatin phase separation. Nature **575**, 390-394. (10.1038/s41586-019-1669-2) 31618757 PMC7039410

[73] Erdel F et al. 2020 Mouse heterochromatin adopts digital compaction states without showing hallmarks of HP1-driven liquid-liquid phase separation. Mol. Cell **78**, 236-249. (10.1016/j.molcel.2020.02.005) 32101700 PMC7163299

[74] Grewal SIS. 2023 The molecular basis of heterochromatin assembly and epigenetic inheritance. Mol. Cell **83**, 1767-1785. (10.1016/j.molcel.2023.04.020) 37207657 PMC10309086

[75] Jorgensen S, Schotta G, Sorensen CS. 2013 Histone H4 lysine 20 methylation: key player in epigenetic regulation of genomic integrity. Nucleic Acids Res. **41**, 2797-2806. (10.1093/nar/gkt012) 23345616 PMC3597678

[76] Lu X, Simon MD, Chodaparambil JV, Hansen JC, Shokat KM, Luger K. 2008 The effect of H3K79 dimethylation and H4K20 trimethylation on nucleosome and chromatin structure. Nat. Struct. Mol. Biol. **15**, 1122-1124. (10.1038/nsmb.1489) 18794842 PMC2648974

[77] Abini-Agbomson S et al. 2023 Catalytic and non-catalytic mechanisms of histone H4 lysine 20 methyltransferase SUV420H1. Biochemistry **83**, 2872-2883. (10.1101/2023.03.17.533220) 37595555

[78] Hong EJ, Villen J, Gerace EL, Gygi SP, Moazed D. 2005 A cullin E3 ubiquitin ligase complex associates with Rik1 and the Clr4 histone H3-K9 methyltransferase and is required for RNAi-mediated heterochromatin formation. RNA Biol. **2**, 106-111. (10.4161/rna.2.3.2131) 17114925

[79] Horn PJ, Bastie JN, Peterson CL. 2005 A Rik1-associated, cullin-dependent E3 ubiquitin ligase is essential for heterochromatin formation. Genes Dev. **19**, 1705-1714. (10.1101/gad.1328005) 16024659 PMC1176008

[80] Jia S, Kobayashi R, Grewal SIS. 2005 Ubiquitin ligase component Cul4 associates with Clr4 histone methyltransferase to assemble heterochromatin. Nat. Cell Biol. **7**, 1007-1013. (10.1038/ncb1300) 16127433

[81] Stirpe A, Guidotti N, Northall SJ, Kilic S, Hainard A, Vadas O, Fierz B, Schalch T. 2021 SUV39 SET domains mediate crosstalk of heterochromatic histone marks. eLife **10**, e62682. (10.7554/eLife.62682) 34524082 PMC8443253

[82] Greenstein RA, Ng H, Barrales RR, Tan C, Braun S, Al-Sady B. 2022 Local chromatin context regulates the genetic requirements of the heterochromatin spreading reaction. PLoS Genet. **18**, e1010201. (10.1371/journal.pgen.1010201) 35584134 PMC9154106

[83] Lewis EB. 1978 A gene complex controlling segmentation in *Drosophila*. Nature **276**, 565-570. (10.1038/276565a0) 103000

[84] Wang H, Wang L, Erdjument-Bromage H, Vidal M, Tempst P, Jones RS, Zhang Y. 2004 Role of histone H2A ubiquitination in Polycomb silencing. Nature **431**, 873-878. (10.1038/nature02985) 15386022

[85] Geisler SJ, Paro R. 2015 Trithorax and Polycomb group-dependent regulation: a tale of opposing activities. Development **142**, 2876-2887. (10.1242/dev.120030) 26329598

[86] Müller J, Kassis JA. 2006 Polycomb response elements and targeting of Polycomb group proteins in Drosophila. Curr. Opin. Genet. Dev. **16**, 476-484. (10.1016/j.gde.2006.08.005) 16914306

[87] Simon J, Chiang A, Bender W, Shimell MJ, O'connor M. 1993 Elements of the Drosophila Bithorax complex that mediate repression by polycomb group products. Dev. Biol. **158**, 131-144. (10.1006/dbio.1993.1174) 8101171

[88] Mendenhall EM, Koche RP, Truong T, Zhou VW, Issac B, Chi AS, Ku M, Bernstein BE. 2010 GC-rich sequence elements recruit PRC2 in mammalian ES cells. PLoS Genet. **6**, e1001244. (10.1371/journal.pgen.1001244) 21170310 PMC3000368

[89] Blackledge NP et al. 2014 Variant PRC1 complex-dependent H2A ubiquitylation drives PRC2 recruitment and polycomb domain formation. Cell **157**, 1445-1459. (10.1016/j.cell.2014.05.004) 24856970 PMC4048464

[90] Veronezi GMB, Ramachandran S. 2023 Nucleation and spreading rejuvenate polycomb domains every cell cycle. *Biorxiv* 2022. (10.1101/2022.08.02.502476)

[91] Lee H-G, Kahn TG, Simcox A, Schwartz YB, Pirrotta V. 2015 Genome-wide activities of Polycomb complexes control pervasive transcription. Genome Res. **25**, 1170-1181. (10.1101/gr.188920.114) 25986499 PMC4510001

[92] Lee C-H et al. 2018 Allosteric activation dictates PRC2 activity independent of its recruitment to chromatin. Mol. Cell **70**, 422-434. (10.1016/j.molcel.2018.03.020) 29681499 PMC5935545

[93] Lee C-H et al. 2019 Automethylation of PRC2 promotes H3K27 methylation and is impaired in H3K27M pediatric glioma. Genes Dev. **33**, 1428-1440. (10.1101/gad.328773.119) 31488577 PMC6771381

[94] Jain SU et al. 2020 H3 K27M and EZHIP Impede H3K27-methylation spreading by inhibiting allosterically stimulated PRC2. Mol. Cell **80**, 726-735. (10.1016/j.molcel.2020.09.028) 33049227 PMC7680438

[95] Lee C-H et al. 2018 Distinct stimulatory mechanisms regulate the catalytic activity of polycomb repressive complex 2. Mol. Cell **70**, 435-448. (10.1016/j.molcel.2018.03.019) 29681498 PMC5949877

[96] Poepsel S, Kasinath V, Nogales E. 2018 Cryo-EM structures of PRC2 simultaneously engaged with two functionally distinct nucleosomes. Nat. Struct. Mol. Biol. **25**, 154-162. (10.1038/s41594-018-0023-y) 29379173 PMC5805599

[97] Yuan W et al. 2012 Dense chromatin activates Polycomb repressive complex 2 to regulate H3 lysine 27 methylation. Science **337**, 971-975. (10.1126/science.1225237) 22923582

[98] Liu C, Yu J, Song A, Wang M, Hu J, Chen P, Zhao J, Li G. 2023 Histone H1 facilitates restoration of H3K27me3 during DNA replication by chromatin compaction. Nat. Commun. **14**, 4081. (10.1038/s41467-023-39846-y) 37429872 PMC10333366

[99] Pirrotta V, Li H-B. 2012 A view of nuclear polycomb bodies. Curr. Opin. Genet. Dev. **22**, 101-109. (10.1016/j.gde.2011.11.004) 22178420 PMC3329586

[100] Boyle S, Flyamer IM, Williamson I, Sengupta D, Bickmore WA, Illingworth RS. 2020 A central role for canonical PRC1 in shaping the 3D nuclear landscape. Genes Dev. **34**, 931-949. (10.1101/gad.336487.120) 32439634 PMC7328521

[101] Rhodes JDP et al. 2020 Cohesin disrupts polycomb-dependent chromosome interactions in embryonic stem cells. Cell Rep. **30**, 820-835. (10.1016/j.celrep.2019.12.057) 31968256 PMC6988126

[102] Francis NJ, Kingston RE, Woodcock CL. 2004 Chromatin compaction by a Polycomb Group Protein complex. Science **306**, 1574-1577. (10.1126/science.1100576) 15567868

[103] Isono K et al. 2013 SAM domain polymerization links subnuclear clustering of PRC1 to gene silencing. Dev. Cell **26**, 565-577. (10.1016/j.devcel.2013.08.016) 24091011

[104] Kundu S, Ji F, Sunwoo H, Jain G, Lee JT, Sadreyev RI, Dekker J, Kingston RE. 2017 Polycomb Repressive complex 1 generates discrete compacted domains that change during differentiation. Mol. Cell **65**, 432-446. (10.1016/j.molcel.2017.01.009) 28157505 PMC5421375

[105] Taherbhoy AM, Huang OW, Cochran AG. 2015 BMI1-RING1B is an autoinhibited RING E3 ubiquitin ligase. Nat. Commun. **6**, 7621. (10.1038/ncomms8621) 26151332

[106] Tatavosian R et al. 2019 Nuclear condensates of the Polycomb protein chromobox 2 (CBX2) assemble through phase separation. J. Biol. Chem. **294**, 1451-1463. (10.1074/jbc.RA118.006620) 30514760 PMC6364756

[107] Kim CA, Gingery M, Pilpa RM, Bowie JU. 2002 The SAM domain of polyhomeotic forms a helical polymer. Nat. Struct. Biol. **9**, 453-457. (10.1038/nsb802) 11992127

[108] Lo SM, Follmer NE, Lengsfeld BM, Madamba EV, Seong S, Grau DJ, Francis NJ. 2012 A bridging model for persistence of a polycomb group protein complex through DNA replication *in vitro*. Mol. Cell **46**, 784-796. (10.1016/j.molcel.2012.05.038) 22749399 PMC3389374

[109] Eeftens JM, Kapoor M, Michieletto D, Brangwynne CP. 2021 Polycomb condensates can promote epigenetic marks but are not required for sustained chromatin compaction. Nat. Commun. **12**, 5888. (10.1038/s41467-021-26147-5) 34620850 PMC8497513

[110] Gray F et al. 2016 BMI1 regulates PRC1 architecture and activity through homo- and hetero-oligomerization. Nat. Commun. **7**, 13343. (10.1038/ncomms13343) 27827373 PMC5105191

[111] Plys AJ, Davis CP, Kim J, Rizki G, Keenen MM, Marr SK, Kingston RE. 2019 Phase separation of Polycomb-repressive complex 1 is governed by a charged disordered region of CBX2. Genes Dev. **33**, 799-813. (10.1101/gad.326488.119) 31171700 PMC6601514

[112] Grau D et al. 2021 Structures of monomeric and dimeric PRC2:EZH1 reveal flexible modules involved in chromatin compaction. Nat. Commun. **12**, 714. (10.1038/s41467-020-20775-z) 33514705 PMC7846606

[113] Jiao L, Liu X. 2015 Structural basis of histone H3K27 trimethylation by an active polycomb repressive complex 2. Science **350**, aac4383. (10.1126/science.aac4383) 26472914 PMC5220110

[114] Kasinath V, Faini M, Poepsel S, Reif D, Feng XA, Stjepanovic G, Aebersold R, Nogales E. 2018 Structures of human PRC2 with its cofactors AEBP2 and JARID2. Science **359**, 940-944. (10.1126/science.aar5700) 29348366 PMC5840869

[115] Mylne JS, Barrett L, Tessadori F, Mesnage S, Johnson L, Bernatavichute YV, Jacobsen SE, Fransz P, Dean C. 2006 LHP1, the *Arabidopsis* homologue of HETEROCHROMATIN PROTEIN1, is required for epigenetic silencing of FLC. Proc. Natl Acad. Sci. USA **103**, 5012-5017. (10.1073/pnas.0507427103) 16549797 PMC1458786

[116] Liu Y et al. 2022 Structural basis for the recognition of methylated histone H3 by the *Arabidopsis* LHP1 chromodomain. J. Biol. Chem. **298**, 101623. (10.1016/j.jbc.2022.101623) 35074427 PMC8861120

[117] Zhang X, Germann S, Blus BJ, Khorasanizadeh S, Gaudin V, Jacobsen SE. 2007 The *Arabidopsis* LHP1 protein colocalizes with histone H3 Lys27 trimethylation. Nat. Struct. Mol. Biol. **14**, 869-871. (10.1038/nsmb1283) 17676062

[118] Veluchamy A et al. 2016 LHP1 regulates H3K27me3 spreading and shapes the three-dimensional conformation of the arabidopsis genome. PLoS ONE **11**, e0158936. (10.1371/journal.pone.0158936) 27410265 PMC4943711

[119] Yang H, Berry S, Olsson TSG, Hartley M, Howard M, Dean C. 2017 Distinct phases of Polycomb silencing to hold epigenetic memory of cold in Arabidopsis. Science **357**, 1142-1145(10.1126/science.aan1121) 28818969

[120] Matsui T, Leung D, Miyashita H, Maksakova IA, Miyachi H, Kimura H, Tachibana M, Lorincz MC, Shinkai Y. 2010 Proviral silencing in embryonic stem cells requires the histone methyltransferase ESET. Nature **464**, 927-931. (10.1038/nature08858) 20164836

[121] Rowe HM et al. 2010 KAP1 controls endogenous retroviruses in embryonic stem cells. Nature **463**, 237-240. (10.1038/NATURE08674) 20075919

[122] Schultz DC, Ayyanathan K, Negorev D, Maul GG, Rauscher FJ. 2002 SETDB1: a novel KAP-1-associated histone H3, lysine 9-specific methyltransferase that contributes to HP1-mediated silencing of euchromatic genes by KRAB zinc-finger proteins. Genes Dev. **16**, 919-932. (10.1101/gad.973302) 11959841 PMC152359

[123] Ivanov AV et al. 2007 PHD domain-mediated E3 ligase activity directs intramolecular sumoylation of an adjacent bromodomain required for gene silencing. Mol. Cell **28**, 823-837. (10.1016/J.MOLCEL.2007.11.012) 18082607 PMC4348069

[124] Iyengar S, Farnham PJ. 2011 KAP1 protein: an enigmatic master regulator of the genome. J. Biol. Chem. **286**, 26 267-26 276. (10.1074/JBC.R111.252569) PMC3143589 21652716

[125] Zeng L, Yap KL, Ivanov AV, Wang X, Mujtaba S, Plotnikova O, Rauscher FJ, Zhou MM. 2008 Structural insights into human KAP1 PHD finger-bromodomain and its role in gene silencing. Nat. Struct. Mol. Biol. **15**, 626-633. (10.1038/NSMB.1416) 18488044 PMC3331790

[126] Ecco G et al. 2016 Transposable elements and their KRAB-ZFP controllers regulate gene expression in adult tissues. Dev. Cell **36**, 611-623. (10.1016/J.DEVCEL.2016.02.024) 27003935 PMC4896391

[127] Wolf D, Goff SP. 2009 Embryonic stem cells use ZFP809 to silence retroviral DNAs. Nature **458**, 1201-1204. (10.1038/NATURE07844) 19270682 PMC2676211

[128] Groner AC, Meylan S, Ciuffi A, Zangger N, Ambrosini G, Dénervaud N, Bucher P, Trono D. 2010 KRAB-zinc finger proteins and KAP1 can mediate long-range transcriptional repression through heterochromatin spreading. PLoS Genet. **6**, e1000869. (10.1371/JOURNAL.PGEN.1000869) 20221260 PMC2832679

[129] Rowe HM, Friedli M, Offner S, Verp S, Mesnard D, Marquis J, Aktas T, Trono D. 2013 De novo DNA methylation of endogenous retroviruses is shaped by KRAB-ZFPs/KAP1 and ESET. Dev. Camb. Engl. **140**, 519-529. (10.1242/dev.087585) PMC4892343 23293284

[130] Tchasovnikarova IA, Timms RT, Matheson NJ, Wals K, Antrobus R, Gottgens B, Dougan G, Dawson MA, Lehner PJ. 2015 Epigenetic silencing by the HUSH complex mediates position-effect variegation in human cells. Science **348**, 1481-1485. (10.1126/science.aaa7227) 26022416 PMC4487827

[131] Thompson PJ, Dulberg V, Moon KM, Foster LJ, Chen C, Karimi MM, Lorincz MC. 2015 hnRNP K coordinates transcriptional silencing by SETDB1 in embryonic stem cells. PLoS Genet. **11**, e1004933. (10.1371/JOURNAL.PGEN.1004933) 25611934 PMC4303303

[132] Kourmouli N, Sun YM, Van Der Sar S, Singh PB, Brown JP. 2005 Epigenetic regulation of mammalian pericentric heterochromatin *in vivo* by HP1. Biochem. Biophys. Res. Commun. **337**, 901-907. (10.1016/J.BBRC.2005.09.132) 16213461

[133] Maksakova IA, Goyal P, Bullwinkel J, Brown JP, Bilenky M, Mager DL, Singh PB, Lorincz MC. 2011 H3K9me3-binding proteins are dispensable for SETDB1/H3K9me3-dependent retroviral silencing. Epigenetics Chromatin **4**, 1-8. (10.1186/1756-8935-4-12) 21774827 PMC3169442

[134] Maksakova IA, Thompson PJ, Goyal P, Jones SJM, Singh PB, Karimi MM, Lorincz MC. 2013 Distinct roles of KAP1, HP1 and G9a/GLP in silencing of the two-cell-specific retrotransposon MERVL in mouse ES cells. Epigenetics Chromatin **6**, 1-6. (10.1186/1756-8935-6-15) 23735015 PMC3682905

[135] Douse CH et al. 2020 TASOR is a pseudo-PARP that directs HUSH complex assembly and epigenetic transposon control. Nat. Commun. **11**, 4940. (10.1038/s41467-020-18761-6) 33009411 PMC7532188

[136] Douse CH, Bloor S, Liu Y, Shamin M, Tchasovnikarova IA, Timms RT, Lehner PJ, Modis Y. 2018 Neuropathic MORC2 mutations perturb GHKL ATPase dimerization dynamics and epigenetic silencing by multiple structural mechanisms. Nat. Commun. **9**, 651. (10.1038/s41467-018-03045-x) 29440755 PMC5811534

[137] Tchasovnikarova IA, Timms RT, Douse CH, Roberts RC, Dougan G, Kingston RE, Modis Y, Lehner PJ. 2017 Hyperactivation of HUSH complex function by Charcot-Marie-Tooth disease mutation in MORC2. Nat. Genet. **49**, 1035-1044. (10.1038/ng.3878) 28581500 PMC5493197

[138] Li J et al. 2011 Structural basis for specific binding of human MPP8 chromodomain to histone H3 methylated at lysine 9. PLoS ONE **6**, e25104. (10.1371/journal.pone.0025104) 22022377 PMC3192050

[139] Timms RT, Tchasovnikarova IA, Antrobus R, Dougan G, Lehner PJ. 2016 ATF7IP-mediated stabilization of the histone methyltransferase SETDB1 is essential for heterochromatin formation by the HUSH complex. Cell Rep. **17**, 653-659. (10.1016/J.CELREP.2016.09.050) 27732843 PMC5081395

[140] Tsusaka T et al. 2018 Tri-methylation of ATF7IP by G9a/GLP recruits the chromodomain protein MPP8. Epigenetics Chromatin **11**, 56. (10.1186/s13072-018-0231-z) 30286792 PMC6172828

[141] Chang Y et al. 2011 MPP8 mediates the interactions between DNA methyltransferase Dnmt3a and H3K9 methyltransferase GLP/G9a. Nat. Commun. **2**, 533. (10.1038/ncomms1549) 22086334 PMC3286832

[142] Liu N, Lee CH, Swigut T, Grow E, Gu B, Bassik MC, Wysocka J. 2018 Selective silencing of euchromatic L1s revealed by genome-wide screens for L1 regulators. Nature **553**, 228-232. (10.1038/nature25179) 29211708 PMC5774979

[143] Robbez-Masson L et al. 2018 The hush complex cooperates with trim28 to repress young retrotransposons and new genes. Genome Res. **28**, 836-845. (10.1101/GR.228171.117) 29728366 PMC5991525

[144] Brothers M, Rine J. 2022 Distinguishing between recruitment and spread of silent chromatin structures in *Saccharomyces cerevisiae*. eLife **11**, e75653. (10.7554/eLife.75653) 35073254 PMC8830885

[145] Moretti P, Freeman K, Coodly L, Shore D. 1994 Evidence that a complex of SIR proteins interacts with the silencer and telomere-binding protein RAP1. Genes Dev. **8**, 2257-2269. (10.1101/gad.8.19.2257) 7958893

[146] Triolo T, Sternglanz R. 1996 Role of interactions between the origin recognition complex and SIR1 in transcriptional silencing. Nature **381**, 251-253. (10.1038/381251a0) 8622770

[147] Boscheron C, Maillet L, Marcand S, Tsai-Pflugfelder M, Gasser SM, Gilson E. 1996 Cooperation at a distance between silencers and proto-silencers at the yeast HML locus. EMBO J. **15**, 2184-2195. (10.1002/j.1460-2075.1996.tb00572.x) 8641284 PMC450142

[148] Ellahi A, Thurtle DM, Genetics JR. 2015 The chromatin and transcriptional landscape of native *Saccharomyces cerevisiae* telomeres and subtelomeric domains. Genetics **200**, 505-521. (10.1534/genetics.115.175711) 25823445 PMC4492376

[149] Hoppe GJ, Tanny JC, Rudner AD, Gerber SA, Danaie S, Gygi SP, Moazed D. 2002 Steps in assembly of silent chromatin in yeast: sir3-independent binding of a Sir2/Sir4 complex to silencers and role for sir2-dependent deacetylation. Mol. Cell Biol. **22**, 4167-4180. (10.1128/MCB.22.12.4167-4180.2002) 12024030 PMC133845

[150] Rusche LN, Kirchmaier AL, Rine J. 2002 Ordered nucleation and spreading of silenced chromatin in *Saccharomyces cerevisiae*. Mol. Biol. Cell **13**, 2207-2222. (10.1091/mbc.e02-03-0175) 12134062 PMC117306

[151] Luo K, Vega-Palas MA, Grunstein M. 2002 Rap1-Sir4 binding independent of other Sir, yKu, or histone interactions initiates the assembly of telomeric heterochromatin in yeast. Genes Dev. **16**, 1528-1539. (10.1101/gad.988802) 12080091 PMC186350

[152] Liou G-G, Tanny JC, Kruger RG, Walz T, Moazed D. 2005 Assembly of the SIR complex and its regulation by O-acetyl-ADP-ribose, a product of NAD-dependent histone deacetylation. Cell **121**, 515-527. (10.1016/j.cell.2005.03.035) 15907466

[153] Oppikofer M, Kueng S, Martino F, Soeroes S, Hancock SM, Chin JW, Fischle W, Gasser SM. 2011 A dual role of H4K16 acetylation in the establishment of yeast silent chromatin. EMBO J. **30**, 2610-2621. (10.1038/EMBOJ.2011.170) 21666601 PMC3155304

[154] Cairns BR, Schlichter A, Erdjument-Bromage H, Tempst P, Kornberg RD, Winston F. 1999 Two functionally distinct forms of the RSC nucleosome-remodeling complex, containing essential AT hook, BAH, and bromodomains. Mol. Cell **4**, 715-723. (10.1016/S1097-2765(00)80382-2) 10619019

[155] Nicolas RH, Goodwin GH. 1996 Molecular cloning of polybromo, a nuclear protein containing multiple domains including five bromodomains, a truncated HMG-box, and two repeats of a novel domain. Gene **175**, 233-240. (10.1016/0378-1119(96)82845-9) 8917104

[156] Buchberger JR, Onishi M, Li G, Seebacher J, Rudner AD, Gygi SP, Moazed D. 2008 Sir3-nucleosome interactions in spreading of silent chromatin in *Saccharomyces cerevisiae*. Mol. Cell. Biol. **28**, 6903-6918. (10.1128/MCB.01210-08) 18794362 PMC2573294

[157] Onishi M, Liou G-G, Buchberger JR, Walz T, Moazed D. 2007 Role of the conserved Sir3-BAH domain in nucleosome binding and silent chromatin assembly. Mol. Cell **28**, 1015-1028. (10.1016/j.molcel.2007.12.004) 18158899

[158] Oppikofer M, Kueng S, Keusch JJ, Hassler M, Ladurner AG, Gut H, Gasser SM. 2013 Dimerization of Sir3 via its C-terminal winged helix domain is essential for yeast heterochromatin formation. EMBO J. **32**, 437-449. (10.1038/EMBOJ.2012.343) 23299941 PMC3567499

[159] Behrouzi R, Lu C, Currie MA, Jih G, Iglesias N, Moazed D. 2016 Heterochromatin assembly by interrupted Sir3 bridges across neighboring nucleosomes. eLife **5**, e17556. (10.7554/eLife.17556) 27835568 PMC5106214

[160] Blander G, Guarente L. 2004 The Sir2 family of protein deacetylases. Annu. Rev. Biochem.**73**, 417-435. (10.1146/annurev.biochem.73.011303.073651) 15189148

[161] Borra MT, O'neill FJ, Jackson MD, Marshall B, Verdin E, Foltz KR, Denu JM. 2002 Conserved enzymatic production and biological effect of O-acetyl-ADP-ribose by silent information regulator 2-like NAD+-dependent deacetylases. J. Biol. Chem. **277**, 12 632-12 641. (10.1074/jbc.M111830200) 11812793

[162] Tung SY, Hong JY, Walz T, Moazed D, Liou GG. 2012 Chromatin affinity-precipitation using a small metabolic molecule: its application to analysis of O-acetyl-ADP-ribose. Cell. Mol. Life Sci. **69**, 641-650. (10.1007/S00018-011-0771-X) 21796450 PMC3266462

[163] Martino F et al. 2009 Reconstitution of yeast silent chromatin: multiple contact sites and O-AADPR binding load SIR complexes onto nucleosomes *in vitro*. Mol. Cell **33**, 323-334. (10.1016/j.molcel.2009.01.009) 19217406

[164] Tung SY, Wang SH, Lee SP, Tsai SP, Shen HH, Chen FJ, Wu YY, Hsiao SP, Liou GG. 2017 Modulations of SIR-nucleosome interactions of reconstructed yeast silent pre-heterochromatin by O-acetyl-ADP-ribose and magnesium. Am. Soc. Cell Biol. **28**, 381-386. (10.1091/mbc.E16-06-0359) PMC5341722 27932495

[165] Wang L et al. 2019 Histone modifications regulate chromatin compartmentalization by contributing to a phase separation mechanism. Mol. Cell. **76**, 646-659. (10.1016/j.molcel.2019.08.019) 31543422

[166] Connelly JJ, Yuan P, Hsu H-C, Li Z, Xu R-M, Sternglanz R. 2006 Structure and function of the *Saccharomyces cerevisiae* Sir3 BAH domain. Mol. Cell Biol. **26**, 3256-3265. (10.1128/MCB.26.8.3256-3265.2006) 16581798 PMC1446965

[167] Neuwald AF, Aravind L, Spouge JL, Koonin EV. 1999 AAA+: a class of chaperone-like ATPases associated with the assembly, operation, and disassembly of protein complexes. Genome Res. **9**, 27-43. (10.1101/gr.9.1.27) 9927482

[168] Chou CC, Li YC, Gartenberg MR. 2008 Bypassing Sir2 and O-acetyl-ADP-ribose in transcriptional silencing. Mol. Cell **31**, 650-659. (10.1016/j.molcel.2008.06.020) 18775325 PMC2696193

[169] Aparicio OM, Billington BL, Gottschling DE. 1991 Modifiers of position effect are shared between telomeric and silent mating-type loci in *S. cerevisiae*. Cell **66**, 1279-1287. (10.1016/0092-8674(91)90049-5) 1913809

[170] Johnson LM, Fisher-Adams G, Grunstein M. 1992 Identification of a non-basic domain in the histone H4 N-terminus required for repression of the yeast silent mating loci. EMBO J. **11**, 2201-2209. (10.1002/j.1460-2075.1992.tb05279.x) 1600945 PMC556687

[171] Tanny JC, Kirkpatrick DS, Gerber SA, Gygi SP, Moazed D. 2004 Budding yeast silencing complexes and regulation of sir2 activity by protein-protein interactions. Mol. Cell Biol. **24**, 6931-6946. (10.1128/MCB.24.16.6931-6946.2004) 15282295 PMC479720

[172] Raiymbek G, An S, Khurana N, Gopinath S, Larkin A, Biswas S, Trievel RC, Cho U-S, Ragunathan K. 2020 An H3K9 methylation-dependent protein interaction regulates the non-enzymatic functions of a putative histone demethylase. eLife **9**, e53155. (10.7554/eLife.53155) 32195666 PMC7192584

[173] Zofall M, Grewal SI. 2006 Swi6/HP1 recruits a JmjC domain protein to facilitate transcription of heterochromatic repeats. Mol. Cell **22**, 681-692. (10.1016/j.molcel.2006.05.010) 16762840

